# Music-based research in neonatal intensive care units requires guidelines grounded in developmental neurology and responsive to the individual needs of preterm infants

**DOI:** 10.3389/fpsyg.2026.1730120

**Published:** 2026-06-03

**Authors:** Kaisamari Kostilainen

**Affiliations:** 1 Cognitive Brain Research Unit, Department of Psychology, Faculty of Medicine, University of Helsinki, Helsinki, Finland; 2 Centre of Excellence in Music, Mind, Body, and Brain, Department of Education, Faculty of Educational Sciences, University of Helsinki, Helsinki, Finland

**Keywords:** developmental care, music intervention, music medicine, music therapy, neonatal intensive care unit, preterm birth, preterm infant

## Abstract

Music-based research involving preterm infants is increasingly being conducted in neonatal intensive care units (NICUs). The objectives and methods of such research should be grounded in developmental neurology and the individual needs of preterm infants. However, existing research in this field is heterogeneous, and there is neither a unified developmental framework nor well-established guidelines to ensure the use of age-appropriate methods to prevent excessive sensory stimulation in this vulnerable population. This perspective article highlights key issues to consider when planning and conducting music interventions in NICUs and emphasizes the urgent need to establish general guidelines to ensure safe and ethical conduct of research. Such guidelines should follow developmental care principles, including risk management to avoid overstimulation, and consider the individual capacities of preterm infants to receive and process stimuli.

## Introduction

The potential benefits of music interventions for preterm infants have gained increasing attention over the past decade, and music-based research in neonatal intensive care units (NICUs) has grown rapidly. Although more studies are being conducted in NICUs, the implementation of music without sufficient background knowledge of developmental factors raises concerns. Existing literature is heterogeneous, with considerable variation in study design, outcome measures, and the extent to which infants’ developmental stage is considered (Costa et al., 2022; Di Fiore et al., 2024; Foroushani et al., 2020; Haslbeck et al., 2023). This indicates that there is no clearly established developmental framework or consensus on standardized practices.

Music-based research conducted in NICUs can be classified as music therapy or music medicine. In music therapy studies, the research questions focus on examining the effects of music therapy, with the intervention conducted by a trained music therapist (Mohan et al., 2021; Yue et al., 2021). In contrast, music medicine studies usually focus on the effects of recorded music in various medical settings, typically without the involvement of a trained music therapist (e.g., Lordier et al., 2019; van der Straaten et al., 2025). However, recent systematic reviews have also shown that live music has been used in music medicine studies and that the clinical expertise of those providing the music stimuli varies (Costa et al., 2022; Foroushani et al., 2020). In addition, there has been ambiguity in the literature regarding the correct use of terminology, as some studies have been reported as music therapy even though the interventions included recorded music without a trained music therapist involved (Ak et al., 2015; Aydin and Yildiz, 2012; Corrigan et al., 2020; Keidar et al., 2014; Shabani et al., 2016). This further highlights inconsistencies in the field, which may lead to confusion and reduced credibility.

Regardless of the approach, conducting music interventions in NICUs requires appropriate expertise to ensure the selection and implementation of safe methods. Currently, no international recommendations on the use of music-based methods in NICU studies are available. The American Academy of Pediatrics, via its Committee on Environmental Health, has provided general guidelines on appropriate sound limits for the NICU environment (<45 dB; American Academy of Pediatrics, Committee on Environmental Health, 1997), and these recommendations have been further developed and updated to provide guidance on creating acoustically suitable units (Balk et al., 2023; White et al., 2013). Some hospital guidelines exist for patient education on the use of parental voice and music (Kostilainen, 2023; Massachusetts General Hospital, 2023), and several publications also describe established music therapy practices (Erdei et al., 2024; Haslbeck and Bassler, 2018, 2020; Loewy, 2015) and clinical guidelines for using early parental vocal contact in the NICU (Filippa and Kuhn, 2024). However, no specific guidelines exist for selecting appropriate music-based methods in studies involving this vulnerable population.

Considering that an increasing number of music intervention studies are conducted in the absence of trained music therapists, evidence-based guidelines grounded in developmental neurology are urgently needed to ensure safe and ethical implementation of music-based research in NICUs. This article highlights key factors to consider and proposes directions for future research based on the developmental care framework and individual developmental needs of preterm infants.

## Music-based research in NICUs grounded in developmental neurology

### Neuroprotection and developmental care for preterm infants

A preterm infant, born before the 37th week of pregnancy (World Health Organization, 2023), is immature and cannot process sensory information in the same way as a full-term infant (Maitre et al., 2020). Prematurity results in an underdeveloped nervous system, which can cause hypersensitivity to sensory input (Lammertink et al., 2021). Preterm births are categorized by gestational weeks (GW) as moderately to late preterm (32–37 GW), very preterm (28–32 GW), or extremely preterm (<28 GW) (World Health Organization, 2023). The gestational age at the time of the study affects how well infants tolerate stimuli, as preterm infants vary in their ability to receive and process external input depending on their developmental level (Di Fiore et al., 2024; Graven and Browne, 2008a).

After preterm birth, infants are exposed to a noxious sensory environment in the NICU, which can hinder neurological development and induce physiological stress (Chang and Merzenich, 2003; Cheong et al., 2020; Rand and Lahav, 2013). Regarding auditory development, preterm infants require protection from excessive noise while also being exposed to positive auditory stimulation to promote neurodevelopment (McMahon et al., 2012; Olejnik and Lehman, 2018). Neuroprotection focuses on regulating the NICU environment to protect the developing nervous system, including minimizing pain, stress, bright lights, and noise (Altimier and Phillips, 2016; Phillips et al., 2024), while neurodevelopmental care aims to support the maturation of the nervous system through appropriate sensory stimulation (Als et al., 1996, 2004; Velasco Arias et al., 2025).

Developmental care, which is widely used in NICUs worldwide, focuses on interpreting infant behavioral cues and responding to them in ways that protect individual developmental trajectories (Als, 1982, 1986, 1998; Soleimani et al., 2020; Vittner et al., 2025). The core aspects of this kind of infant-driven care involve each infant’s abilities to interact and receive stimuli, their need for rest, and their ways of communicating these needs. Family-centered care, in which families are active participants from the outset, is an integral part of developmental care (Als, 1998; Hastuti et al., 2026; Velasco Arias et al., 2025). Music interventions in NICUs should be designed to align with a developmental care framework and to be responsive to each infant’s developmental needs.

### Practical implications for music-based research in NICUs

#### Methodological considerations

When developmental neurology and care principles are translated into music-based research, several methodological considerations become critical. Infants born preterm are vulnerable not only because of their immaturity, but also because of possible medical conditions and their associated treatments (e.g., World Health Organization, 2023; Lee et al., 2020; Teune et al., 2011). Hence, only clinically stable preterm infants should be included in music interventions (cf. Epstein et al., 2021).

Preterm infants differ in their communicative abilities compared to full-term infants, as their neurobehavioral organization and regulatory capacities are less developed (Als, 1982; Als et al., 2005; Eckerman et al., 1994; Pineda et al., 2013, 2022). A substantial amount of energy is expended on feeding, growth, and processing sensory input, which may occasionally lead to fatigue and reduced tolerance for external stimulation. Signs of fatigue or overstimulation can include, but are not limited to, fast breathing, yawning, finger splay, sudden movements, startles, floppy limbs, agitation, or falling asleep suddenly (Als, 1986; Giganti et al., 2002; Peng et al., 2013). When such signs are observed, social interaction and external stimulation should be paused to allow the infant to rest and regain strength. When preterm infants are ready to interact and the stimuli provided are appropriate for their level of development, they breathe steadily, their limb movements are smooth, and they observe faces and objects calmly and alertly (Als, 1986; Montirosso et al., 2012; Vittner et al., 2025).

All preterm infants are unique, with individual developmental needs and ways of communicating. Consistent with the developmental care framework, the cues of preterm infants should be read continuously and reciprocated appropriately (Als, 1982, 1986, 1998; Montirosso et al., 2012; Vittner et al., 2025). This also applies when conducting music studies, where signs of stress must be monitored for the duration of sound exposure. It is impossible to determine in advance exactly how much and what kind of stimulus is appropriate at each gestational age (Altimier and Phillips, 2016; Peng et al., 2013). For this reason, prioritizing interaction-based methods in music interventions would be essential, allowing each infant’s reactions to be interpreted and responded to in real time.

#### Considerations for the developmental appropriateness of auditory stimuli

Developmentally supportive stimuli are calm, simple, and predictable, as sudden changes in the auditory environment can cause a stress response and disrupt self-regulation, leading to unstable heart rate, breathing, and oxygen saturation (Als, 1986; Graven, 2000; Kuhn et al., 2012). For example, hearing parental voices (speech or singing) after preterm birth provides biologically meaningful auditory input that supports self-regulation, pain management, and promotes neurodevelopment (Filippa et al., 2017, 2021; Filippa and Kuhn, 2024; Kostilainen et al., 2021b; Partanen et al., 2022; Travis et al., 2025). Infant-directed singing using simple and repetitive melodies offers a sensitive way to interact with hospitalized infants (Filippa et al., 2017; Haslbeck and Bassler, 2018, 2020; Kriechbaum et al., 2025). In addition, trained music therapists can use NICU-appropriate instruments played softly at suitable frequency and volume levels (e.g., Bertsch et al., 2020; Haslbeck and Bassler, 2018, 2020; Loewy et al., 2013) to provide interactive stimuli tailored to each infant’s developmental level (e.g., Haslbeck and Bassler, 2018, 2020; Loewy, 2015).

Because pre-recorded music cannot be modified during the intervention or tailored to meet each infant’s individual needs, its use is problematic due to the increased risk of overstimulation. If recorded music is used, stimuli should be selected based on gestational age and individual auditory processing capacity. Calm, soothing, and simple stimuli would be generally most appropriate for the NICU environment, taking into consideration developmental care principles. According to Graven (2000) and the recommendations of the NICU Sound Study Group and Expert Review Panel, headphones, where music is played directly to preterm infants’ ears, should not be used, which is a concern also raised by Vitale et al. (2021). Furthermore, using audio recordings should be brief and should not be left unattended with high-risk infants (Graven, 2000), a principle that is also supported by more recent work (Mikulis et al., 2021). Thus, a researcher or nurse must be present to monitor the infant’s reactions throughout the music intervention. If there are any signs of overstimulation and the infant communicates that it needs a change, the intervention must be discontinued (Als, 1986; Als et al., 2004; Montirosso et al., 2012; Vittner et al., 2025). It is also important to note that recordings should not be used as a substitute for hearing speech in live interaction, preferably from a parent or caregiver [see Filippa and Kuhn (2024) and Graven (2000)].

#### Neurobiological perspectives on music processing and considerations for research

Given the immaturity of neural mechanisms, as outlined above, particular caution is required when introducing preterm infants to stimuli such as music. Music is a complex auditory input consisting of multiple acoustic and musical elements, including rhythm, melody, tempo, timbre, and harmony. These elements are already present prenatally, such as pulse (e.g., heartbeat, respiration) and pitch contour (e.g., maternal voice) (Provasi et al., 2021; Teie, 2016), which may provide preterm infants with biologically meaningful continuity after birth. However, because maternal tissues and amniotic fluid no longer protect the vulnerable, rapidly developing brain by providing acoustic filtering after preterm birth, music used in the NICU should be carefully selected (Di Fiore et al., 2024; Graven, 2000; Graven and Browne, 2008a, 2008b).

Exposure should begin with simple and brief sounds, as prolonged and complex input can be exhausting for preterm infants at lower gestational ages (Als, 1982, 1986, 1998; Graven, 2000; Vittner et al., 2025). Even sensory input that is potentially beneficial can become harmful if presented at an inappropriate developmental stage or at excessive intensity (Graven, 2000; Graven and Browne, 2008a). Accordingly, whether developmental benefits are achieved depends on the type of auditory stimulus and duration of the exposure at each gestational age (Di Fiore et al., 2024). Therefore, stimuli should be offered carefully and only to the extent that the infant remains calm and receptive. In line with developmental care principles, the amount and complexity of stimuli can be gradually increased as the infant communicates behavioral and physiological readiness (Altimier and Phillips, 2016; Als, 1982, 1998; Di Fiore et al., 2024; Vittner et al., 2025).

Major structures of the inner ear develop by 23–25 GW, and from around 25 GW the cochlear nerve begins transmitting sound-evoked signals to the auditory cortex (Clark-Gambelunghe and Clark, 2015; Graven and Browne, 2008b; Hall, 2000). However, the capacity to process and habituate to sounds, and to regulate sensory information in the environment, becomes increasingly mature from approximately 32 GW onwards (Bellieni et al., 2005; Groome et al., 1993; Morokuma et al., 2004). Furthermore, homeostasis, defined as the physiological and behavioral regulation of the body, can be more effectively maintained only after sufficient maturation of the autonomic nervous system (de Souza Filho et al., 2019; Lucchini et al., 2016). As the infant’s ability to process complicated sensory information, such as music, and to maintain homeostasis is limited before 32 GW, both gestational age and the type of stimuli used in studies should be carefully considered.

When examining the effects of recordings, it is essential to consider a developmental neurological perspective. Based on the maturational limitations and developmental care principles discussed earlier, preterm infants cannot be treated as purely passive music listeners [cf. Papatzikis et al. (2024); see also Vittner et al. (2025)]. Their incomplete neurological development requires constant regulation of the provided stimulus, taking into account the infant’s current state and sensory load—both of which can vary several times over an intervention. Thus, interaction and real-time assessment should be prioritized in future studies.

Anchoring music-based research in NICUs within these developmental principles provides a foundation for understanding gradual maturational milestones. This perspective explains why stimuli such as music require careful consideration of gestational age, stimulus characteristics, and intervention duration. This approach helps minimize the risk of overstimulation and maximizes the potential for infants to benefit from the stimuli.

#### Challenges and risks of playing music inside incubators

In many studies, classical music recordings have been played inside an incubator (Amini et al., 2013; Aydin and Yildiz, 2012; Keidar et al., 2014; Lafferty et al., 2023; Lubetzky et al., 2010; Shin et al., 2022; van der Straaten et al., 2025). As high sound-pressure levels can be harmful for preterm infants, there are many factors to consider when playing music inside an incubator. The acoustics of an incubator are complicated and differ from the acoustics in an open space. The hard surfaces of the incubator do not dampen sounds but effectively reflect them back inside (Puyana-Romero et al., 2020, 2021). Therefore, reverberation time, the time it takes for a sound to fade away in an enclosed space after the sound source has stopped, can be especially long in the incubator and is affected by the reverberation time of the room (Puyana-Romero et al., 2020, 2021). Reverberation can make certain frequencies sound louder and sharper in the infant’s ear (Marik et al., 2012; Puyana-Romero et al., 2020, 2021; Restin et al., 2021). Furthermore, resonance frequencies can occur when lower frequencies reverberate in the incubator structures, making them uncomfortably loud (Reuter et al., 2023). Resonance must also be considered outside the incubator; no equipment or instruments should be placed on top (Olejnik and Lehman, 2018), and instruments with certain frequencies should not be used in close proximity to the incubator (Bertsch et al., 2020).

In summary, the acoustics within the small, enclosed space of an incubator are not controllable and therefore, playing music inside the incubator carries risks that cannot be entirely eliminated. To protect the immature nervous system and developing hearing of preterm infants, discussion is needed regarding the safety and necessity of playing music inside incubators. Many studies demonstrate that an incubator’s fan and the heating and humidity systems produce noise that exceeds the recommended levels (Fernández Zacarías et al., 2018; Kent et al., 2022; Parra et al., 2017; Restin et al., 2021), especially when respiratory support is used (Bertsch et al., 2020; Marik et al., 2012; Reuter et al., 2023). Moreover, Kuhn et al. (2012) indicated that even a 5–10 dB change in the signal-to-noise ratio inside the incubator can alter the well-being of very preterm infants. Adding music as a calming factor to this already noisy environment is challenging, as it may increase the overall noise. Therefore, it may be more appropriate to first address the noise generated by incubators and urge their manufacturers to develop quieter and acoustically safer models before adding more stimuli inside.

Since it is known that the incubator masks not only external noise but also developmentally relevant speech sounds (Restin et al., 2021), hearing parental voices is a developmentally appropriate stimulus and could be used briefly when the windows are opened during routine care situations. This approach might offer a safer and developmentally appropriate way to provide auditory stimulation, as (1) infants cared for inside the incubator have not yet achieved mature physiological homeostasis (e.g., Dunne et al., 2024), (2) open incubator windows alter reverberation and resonance characteristics compared with a closed incubator (e.g., Puyana-Romero et al., 2021), and (3) it may reduce hygiene-related concerns, since no additional equipment is placed inside the incubator, in accordance with WHO infection prevention principles (World Health Organization, 2002) and unit-specific NICU infection control policies.

Music medicine studies in which classical music has been played inside the incubator for very preterm infants or infants with a birth weight of 1,000–1,500 g for extended periods of 40–60 min (Aydin and Yildiz, 2012; Keidar et al., 2014; Lubetzky et al., 2010) raise significant concerns regarding developmental appropriateness. According to published reports, these studies have not adequately considered one or more evidence-based factors related to developmental care principles, incubator acoustics, sound-level assessment and measurement practices, and the developmental suitability, including the selection of the stimuli and intervention duration. In some cases, even the so-called “Mozart effect” on very preterm infants was examined (Amini et al., 2013; Keidar et al., 2014; Lafferty et al., 2023; Lubetzky et al., 2010), an approach that is highly questionable from a developmental care perspective, given the maturational limitations and vulnerability of this population. Considering preterm infants’ underdeveloped central and autonomic nervous systems and limited auditory processing capacities (Graven and Browne, 2008b; Kuhn et al., 2012; McMahon et al., 2012), the use of classical or other multi-instrumental compositions with more complex musical elements increases the risk of overstimulation both inside and outside the incubator.

By using methods such as live music therapy, infant-directed singing or humming with simple, repetitive, and predictable musical elements, infants can be soothed and their overall development—including neurodevelopment and behavioral regulation—can be promoted through developmentally appropriate approaches that minimize the risk of overstimulation (Filippa et al., 2017; Haslbeck and Bassler, 2018; Haslbeck et al., 2020; Kostilainen et al., 2021a, 2021b; Partanen et al., 2022). Future studies should carefully consider these issues when planning music interventions for preterm infants.

With increasing postmenstrual age, infants usually demonstrate increasing tolerance to stimuli and an enhanced capacity to interact with their environment (Als, 1982, 1986; Di Fiore et al., 2024; Zandvoort et al., 2024). At this point, when the nervous system is mature enough, and infants can regulate and calm themselves, exposure to more active and diverse music may be developmentally more appropriate. Individual abilities to receive stimuli must nevertheless be considered, as infants born preterm may also experience sensory hypersensitivity at a later stage of development (Niutanen et al., 2019; Ryckman et al., 2017).

#### Choosing appropriate outcome measures

Outcome measures should be carefully selected in NICU music-based research. For example, some studies have used resting energy expenditure (REE) as a primary indicator of relaxation (Keidar et al., 2014; Lubetzky et al., 2010) and heart rate as a primary indicator of self-regulation (Cevasco-Trotter et al., 2019). However, REE and heart rate alone are non-specific indicators of relaxation or self-regulation, as both parameters can also decrease in response to overstimulation. Due to immature autonomic control, sensory stimulation can activate reflexive changes in heart rate, reflecting the complex cardiovascular responses of preterm infants to stimulation (Lagercrantz et al., 1990; Sweeney and Blackburn, 2013). Moreover, REE can decrease during acute physiological stress or illness, as part of the body’s energy-conservation response (Feferbaum et al., 2010), suggesting that reduced REE does not necessarily indicate relaxation or improved well-being. This physiological downregulation has already been described by Als (1982, 1986) in terms of disengagement behavior, i.e., “*tuning out*,” in which the infant may appear calm but is physiologically stressed and withdraws from the situation when regulation of stimuli is no longer possible.

To conclude, using only one physiological outcome measure, such as REE or heart rate, in music intervention studies is insufficient to demonstrate relaxation or self-regulation. Instead, it would be advisable to use several physiological outcome measures that complement each other. When measuring REE or other physiological stress markers, other real-time metrics are needed to detect acute responses, such as heart rate variability (HRV), which measures autonomic nervous system regulation (e.g., Donnellan et al., 2025), or oxygen saturation, which responds dynamically to stress (Peng et al., 2013, 2014) and caregiving (Allinson et al., 2017; Brashear et al., 2023). Another option would be to combine physiological data, such as HRV and oxygen saturation, with systematic behavioral data. Careful and thorough planning of outcome variables in NICU music studies is crucial to correctly interpret the reactions of preterm infants.

## Discussion

The arguments presented in this paper underscore the importance of grounding music-based research in NICUs in developmental neurology, individualized, infant-driven care, and safe acoustic practices. The planning and implementation of music interventions should be based on the developmental needs of preterm infants, with research objectives and methods consistently reflecting this (e.g., Als, 1982; Altimier and Phillips, 2016; Vittner et al., 2025). Considering their different developmental stages and thresholds for auditory input, preterm infants should be treated as individuals rather than as a homogeneous group (e.g., Di Fiore et al., 2024; Graven and Browne, 2008b). In accordance with the principles of developmental care stated above, music stimuli must be age-appropriate, and the infant’s individual tolerance for stimuli and sensory processing capacity needs to be carefully assessed when determining the suitability of stimuli and duration of exposure. For this reason, individually tailored interventions responsive to each infant’s gestational age and neurodevelopmental status are essential for optimizing safety and maximizing the benefits of music interventions.

All researchers conducting music interventions with this vulnerable population must remain aware that music may overstimulate preterm infants and potentially cause harm if applied without consideration of maturational factors (e.g., Als, 1986; Di Fiore et al., 2024; Graven and Browne, 2008a). As discussed in relation to sensory vulnerability and developmental care principles, the use of complex music in the NICU, such as classical compositions, may not always be developmentally appropriate, particularly inside an incubator with limited control over acoustic properties. Instead, consistent with the evidence outlined above, preference should be given to simple, predictable, and calming acoustic features. These may include exposure to live, biologically meaningful parental voices, infant-directed singing, or soothing, repetitive, and simple melodies played on appropriate instruments by a NICU-trained music therapist. Given the strong evidence supporting family-centered care and the benefits of parental voice exposure after preterm birth, the inclusion of parents in interventions is highly recommended (Phillips et al., 2024; Caskey et al., 2014; Chen et al., 2025; Filippa et al., 2017, 2021; O'Brien et al., 2018).

This article does not seek to dismiss the potential benefits of different music interventions but rather to urge caution and ensure that all stimuli are developmentally appropriate for each infant in intensive care. To promote both safety and methodological rigor, studies should be conducted by a multidisciplinary research team, including a developmental care professional and a NICU-trained music therapist. Publications should also provide detailed descriptions of how developmental factors and risk management were addressed.

The author hopes this article will promote discussion on these topics and lead to the establishment of general guidelines for music-based research in NICUs. These guidelines could be developed by an international, multidisciplinary panel of experts, including neonatologists, developmental care professionals, nurses, music therapists with extensive clinical experience in neonatal care, and researchers with expertise in auditory development after preterm birth. Such comprehensive global guidelines are urgently needed, not only to ensure the safe conduct of studies, but also to support the appropriate promotion of non-medical NICU treatments, with the ultimate goal of enhancing the well-being and development of preterm infants.
